# Influencing factors of employee brand equity from the perspective of FinTech

**DOI:** 10.3389/fpsyg.2022.1047936

**Published:** 2022-11-21

**Authors:** Lichao Lin, Ziling Huang, Xiaofei Jia

**Affiliations:** ^1^Faculty of Finance and Economics, Guangdong University of Science and Technology, Dongguan, Guangdong, China; ^2^School of Digital Economics, Dongguan City University, Dongguan, Guangdong, China; ^3^Faculty of Accounting School, Binzhou Polytechnic, Binzhou, Shandong, China

**Keywords:** employee brand equity, FinTech, enterprise, psychological factor, China

## Abstract

Recent years have seen a heated discussion on what influences employees’ recognition of enterprise brand equity among the psychological community. Some empirical evidence suggests that the brand equity of consumers and brand equity of sales are two categories that should be studied. However, more recent discussions have focused more on the former. In contrast, this study discusses the factors influencing brand equity based on the employee level. Moreover, this paper examines whether FinTech can help companies get out of financing difficulties and thus improve employees’ brand equity recognition. This research illustrates how FinTech has become an essential driver of brand equity value. Based on micro panel data on enterprises from 2011 to 2019, we analyze the transmission mechanism between the two factors and the mediating role of financing constraints. FinTech’s development promotes employees’ recognition of corporate brand equity, and financing constraints are an essential transmission path between the two factors. Furthermore, the impact of financing constraints on employee brand equity identity is characterized by cross-regional economic heterogeneity. In contrast, the development of FinTech characterizes the cross-enterprise heterogeneity in employees’ recognition of brand equity value. Altogether, this study demonstrates the promising application of FinTech in influencing the mechanisms of employee brand equity.

## Highlights

– FinTech can not only directly increase the expectation of employee brand equity but also realize this process indirectly. Importantly, financing constraint is an effective intermediary variable between the two.
– The inhibitory effect of financing constraints on employee brand equity is significantly weaker for state-owned enterprises than that for private enterprises.
– The inhibitory effect of financing constraints is significantly stronger for private enterprises in the eastern region than those from other regions.

## Introduction

In the past decade, to mitigate the impact of 2008 subprime mortgage crisis, the Chinese government undertook a 4-trillion-yuan economic stimulus (Lian and Chen, 2021). While this helped create tens of thousands of small- and medium-sized enterprises (SMEs), overly loose regulatory policies left the liberalized market open to a variety of problems caused by the fragile market structure (Yuan et al., 2021). For example, supervising the quality of brand equity among different enterprises was not easy. Therefore, the China Banking Regulatory Commission (CBRC) launched an emergency intervention, following which commercial banks tightened their loose financing policy. However, due to tighter financing approvals, non-state-owned enterprises (non-SOE; e.g., SMEs) struggled in the highly deteriorated financing environment (Lian and Chen, 2021).

Recently, more and more corporate executives have become concerned about the impact of a broken corporate capital chain on the brand equity of their employees (Zhu et al., 2017; Guo et al., 2019). Brand equity can be a tool for quantifying the role of brands (Wang et al., 2020) and serve as an essential element for enterprises to obtain profits and revenues from external markets. Brand equity provides administrators with strategic support by weighing the pros and cons of brands in terms of finance, market competition, and consumption experience. Research shows that employees’ sense of belonging to and identification with corporate brand assets comes from stable salary income from stable corporate cash flow generated by high-quality corporate brand assets (Wei and Xu, 2016; Zhang and Liu, 2018). However, tighter financing constraints can hinder stable operations and cash flow generation and, thus, employee brand equity. FinTech^1^ has emerged as a prominent novel solution to these problems and can become a new lifeline of SMEs and their brand equity. Specifically, it can ease financing constraints for enterprises (Klapper et al., 2019; Yin et al., 2019; Huang et al., 2020). FinTech can be very valuable tool for enhancing brand equity by easing financing constrains. As an essential supplement to the traditional financial system, FinTech is characterized by inclusiveness (Hsu et al., 2014) and remolding (Yang, 2018). It performs a vital role in business development by providing real businesses with finance to perform resource allocation functions (Xue and Hu, 2020). Moreover, the wide application of cutting-edge technologies, such as cloud computing, blockchain, big data, and artificial intelligence, has promoted the rapid rise of the digital finance or FinTech industry. For example, artificial intelligence (He et al., 2022) and enterprise intelligence (Zhang and Li, 2022) may radically transform traditional labor relations between employees and enterprises. Importantly, by improving the financing environment for SMEs, FinTech can revive employees’ confidence in corporate brand assets, and thus, is likely to be an essential factor that affects employee brand equity. Therefore, understanding the relationship between FinTech and employee brand equity, and mechanisms underlying this relationship is important.

The concept of brand equity is the clarity to quantify and measure the strengths and weaknesses of brands at all levels of finance, market competition, and consumer mindset, and to provide decision support for brand managers; that is, different conceptual models providing clarity under differentiated research perspectives. The development of financial technology has penetrated digital logic to all levels of society, including the communication of brands and the formation of brand equity. Given this context, we need to understand how the conceptual model of brand equity needs to adapt to digital changes. Digital brand equity currently exists as a distortion of the traditional model (Wang et al., 2020), which only quantifies the brand-consumer relationship without considering the brand’s digital content and the value of the producer. For example, some studies discuss the impact of product innovativeness (Qu, 2022), market share (Zhang and Ye, 2021), advertising, and R&D investment (Chu and Keh, 2006; Peterson and Jeong, 2010; Mai and Li, 2016) on corporate brand equity enhancement. However, brand equity should not ignore producers’ contribution and emotional devotion. Especially after the addition of FinTech and now social media, the emotional orientation of corporate employees towards brand equity is likely to outweigh the influence of other individual behaviors. Therefore, focusing more on employees’ perception of brand equity and FinTech’s effect on it is an exciting research topic.

Our work differs from the extant literature in the following ways: First, a crucial role of FinTech is how it can affect employee brand equity by adjusting financing constraints. To the best of our knowledge, few studies have compared and analyzed FinTech alone as a variable influencing employee brand equity. Second, we consider how these relationships change with the nature of enterprises’ property rights and which economic region of the country they are located in. Specifically, commercial banks are more inclined to lend to state-owned enterprises (SOE) than private ones, or that some Chinese regions are economically more developed than other regions. Thus, there may be heterogeneity in our results. Considering these factors can be useful for measuring China’s economic development and the capital agglomeration effect of listed companies. The rest of this study proceeds as follows. Section 2 reviews the relevant literature and develops our hypotheses. Section 3 outlines the methodology. Section 4 describes the empirical and robustness check results. Finally, Section 5 presents the conclusions of this study.

## Literature review and theoretical hypotheses

### Literature review

#### Brand equity

The establishment of brand awareness (Zhang et al., 2015) and brand image (Seo and Park, 2018) are symbolic achievements to attract consumers or employees to regard them as their preferred brand. In the past, most relevant studies were based on the two bedding of Customer-Based-Brand Equity (CBBE) and Sales-Based-Brand-Equity (SBBE; Datta et al., 2017). CBBE means the attractiveness of brand names or symbols to consumers, a type of value transfer at the bedding of consumer perception (Godey et al., 2016). For example, Nawi et al. (2022) examined how the social media marketing of a mobile brand affects the brand equity, relationship equity, and value equity of customers. Based on the mediating role of brand equity and brand identity, Farzin et al. (2022) researched the influence of social media marketing and eWOM on consumers’ willingness to pay premiums. Amoako (2022) extends the applicability of brand equity as a mediating variable to enhance the impact of location and service mart availability on purchasing behavior of customers. Researchers agree that these factors and concerning studies have solved some issues in the construction of brand equity value from the perspective of customers. However, these studies still have some limitations. So some other scholars studied from the dimension of SBBE. SBBE refers to the impact of internal brand construction on the brand support of employees. Previously, only early or few studies have revealed the perception of employees in the critical areas of internal brand construction. Therefore, it is significant to study this field further.

#### Employee-based brand equity

The importance of employees in establishing strong brand equity was widely emphasized in past years. Recently, a study by Raj (2020) has reported on how internal brand construction has become a human resources (HR) structure for creating, implementing, and measuring an employee-based brand image in an organization. By interviewing 443 respondents from various industries, researchers analyzed how internal brand strategies help employees improve employee identity with the brand image in terms of psychology, physics, spirit, finance, and social welfare. According to the study by Kahn (1992), employee engagement is a multidimensional index, and employees can get involved in the organization through emotional, cognitive, and physical behaviors. Shuck and Reio (2014) explained the phenomenon that poor labor force participation causes damage to organizations, which means that employees with high participation have a higher happiness index. Besides employee engagement, the investment in employee benefits may also improve productivity and employee morale. A job survey of professionals in Indian IT firms studied the relationship between employee welfare and employment participation (Sivapragasam and Raya, 2018). This study revealed a statistically significant relationship between production and employee incentive.

#### Employee brand equity and salary incentives

Human capital is a core element in an enterprise’s internal governance (Sun et al., 2021). Compensation incentives are vital for top executives to influence technological innovation decisions (Guo et al., 2019). Furthermore, monetary compensation incentives for general employees positively affect R&D investment in listed companies (Tang and Zhen, 2009). These observations are supported by extant research, including research on the level of compensation of general employees (Wang and Liu, 2008; Guo and Lu, 2011; Ye et al., 2013) and executives (Zhang et al., 2003; Ruan et al., 2013; Shi and Cheng, 2022). Interestingly, some find that general employee compensation contributes more significantly to the creation of value added by the enterprise (Zhu et al., 2017). Therefore, salary incentive is an essential dimension in studying enterprise management and brand equity. After then, this paper will apply worker salary as a measure of employee brand equity to conduct the subsequent research.

#### Employee brand equity and FinTech applications

Empirical evidence shows that the development of digital technologies, such as big data and cloud computing, provides application scenarios for digital finance. This can be important for improving the corporate financing environment and promoting corporate innovation development (He and Liu, 2022; Wang and Wang, 2022). Some entrepreneurs are involved in constructing or using FinTech teams to meet the needs of the times and industrial upgrading. That means the management has a reasonable expectation of the prospects of FinTech, which requires meeting two conditions simultaneously: First, the excess return (or expected return) the enterprises earn from developing FinTech should exceed the total R&D investments in it. Second, the ratio of the excess salary paid by the enterprises for FinTech development to the current operating income should be slightly higher than the ratio of the employee salary to the operating income in the previous financial year. This assertion is in line with the fact that the share of social labor income has continued to rise since 2011 (Lu and Tian, 2020).

Meanwhile, naysayers may not see the company’s future in FinTech or perhaps it does not have sufficient and reasonable financial budgets to support FinTech-focused R&D. Still, to avoid competitors encroaching on their market share, managers may passively increase FinTech investments. If the enterprise succeeds in research and development, it will follow the supporters’ path. Otherwise, if the enterprise cannot make ends meet for an extended period, its capital gap may lead to a shrinking market share. In severe cases, it may directly lead to bankruptcy and liquidation. These enterprises cannot continue to operate because it does not meet the goal of no listed company may earn negative income for two consecutive years (Shanghai Stock Exchange Stock Listing Rules (Revised December 2020), page 84–110), thus, we do not discuss them. Therefore, more employee compensation can facilitate the growth of FinTech.^2^

### Theoretical hypotheses

The cash provides security for long-term business activities. Some scholars discussed the impact of financing constraints on firm technological innovation (Ju et al., 2013) and technological R&D (Lian and Su, 2009; Cai et al., 2012) and concluded that financing constraints have a significant inverse effect on the latter. Shao and Hu (2022) note that FinTech affects enterprises’ investment efficiency through two types of mediating variables: “financing constraints and financial expense ratio” and “debt leverage and risk stability.” Lu and Liu (2018) suggest that FinTech innovation (e.g., big data and cloud computing) can translate resource allocation efficiency into real productivity by reducing the search and transmission costs of financial resources, alleviating information asymmetry, and lowering enterprises’ financing costs to achieve this. However, enterprises also need to appropriately compensate FinTech employees to retain them. Given the extensive literature on FinTech and financing constraints (Lu and Liu, 2018; Deng and Zhao, 2022; Shao and Hu, 2022; Wang et al., 2022), we seek to examine how financing constraints affect the relationship between FinTech and enterprise’s brand equity. Specifically, FinTech may mitigate financing constraints, and thus, aid corporate revenue; this enhanced revenue serves as a good proxy for employees’ recognition of corporate brand equity. To verify this conjecture, we need to separately examine the theoretical transmission mechanism between “FinTech and financing constraints” and “financing constraints and employee brand equity.”

From the cross-matrix perspective of FinTech and inclusive finance, Hu and Cheng (2020) argue that FinTech will disrupt and fully penetrate the field of financial inclusion (such as payment and settlement, lending and financing, investment and wealth management, risk management, financial supervision, etc.). On the one hand, financial capital provided by FinTech builds the bridge for industrial restructuring and regional economic linkage (Yang and Zhang, 2018). On the other hand, FinTech provides efficient, convenient, and reliable market-oriented services to the real economy by improving the efficiency of capital allocation and optimizing the asset structure. Intermediaries may pose a significant threat to the financing environment, technology development, etc., of enterprises under the traditional financial operating model. Their excessive market participation can lead to common drawbacks such as opaque information, short time frame, and inefficient marketization (Zhang and Zhao, 2019). However, FinTech can help in overcoming the development dilemma of inclusive finance and alleviating the financing difficulties of SMEs (Huang and Huang, 2018). First, FinTech can effectively reduce the cost of information search and the timely discovery of the intrinsic value of innovation. Second, it can improve the level of innovation output of enterprises by easing financing constraints, and reducing financial expenses and financial leverage (Wang et al., 2019). Finally, it can improve and promote the credit and maturity structure of the credit segment and enhance the level of risk-taking in the banking industry by intensifying interbank competition behavior (Sun et al., 2020). Based on this discussion, the following hypothesis is proposed.

*Hypothesis 1 (H1):* FinTech can significantly decrease the financing constraints of enterprises.

Next, we will discuss the relationship between financing constraints and employee brand equity. Neumeyer and Perri (2005), the earliest proponents of the theory of working capital, believe that rising market interest rate will reduce the borrowing capital, working capital, labor demand, and labor wages of the enterprises, while raising labor income and employee brand equity. Subsequently, inspired by the previous findings, Kabaca (2009) proposed the transmission mechanism of “interest rate → working capital → labor income.” When enterprises need not borrow funds from the market (θ=0), the labor income can be equivalent to the labor output elasticity; otherwise, it has a negative relationship with the market interest rate. However, this hypothesis is applicable only when the sample covers sufficient period and cannot be used to explain the short-term trend of China’s labor income. After that, Aziz and Cui (2007) proposed the indicator φ to reflect the degree of financial easing to represent the labor income, which can reflect employee brand equity (BE) as follows.


(1) $$\;BE=\frac{\varphi \beta }{\gamma }\left[\frac{\gamma }{\beta }-\left(1-\delta \right)+\varphi \right]\;$$

Where β refers to the discount rate, δ refers to the depreciation rate, and γ represents technological progress. Assuming that an enterprise’s entire working capital comes from borrowing and financing, easing represents the ratio between working capital and capital stock. Then, there will be a forward correlation between the degree of financing easing and the size of capital (or labor income). This reveals the positive relationship between financing constraints and brand equity. Zhu and Zhao (2016) propose a mechanism of the impact of financing constraints on the labor income of enterprises based on the idea of “Liquid capital constraints.” Specifically, the enterprise’s working capital decreases as the financing constraint increases, which constrains the allocation of profits to factors of production. Enterprises can only raise working capital through internal savings when faced with external market financing difficulties. However, in the long run, this will hinder the enterprise from appropriately compensating employees. Suppose the capital accumulation equation is $K_{i,t}^{s}=k_{i,t-2}^{s}+l\theta_{i,t-2}^{s}$. Next, consider a condition when the enterprises may encounter financing constraints. For instance, when an enterprise takes out a loan to pay all employees or places a cap on working capital, the total amount of payroll should not exceed the loan limit it can obtain. Note that the limit is subject to the total value of all asset’s collateral. That is:


(2) $$\;w_{t}L_{i,t}^{s}\leq \vartheta K_{i,t}^{s}$$

The enterprises have the goal of profit maximization and are characterized by size preference. We assume that for enterprise i, the price of the product is $P_{i,t}^{s}$; the product demandis $Y_{i,t}^{s}$; the enterprise’s labor capital is $L_{i,t}^{s}$; the enterprise’s size preference is $\theta_{i,t}^{s}$, with $\theta_{i,t}^{s}\in \left[0,1\right]$; the Lagrange multiplier of the credit constraint is λ, with λ>0; the demand elasticity of the product is δ, with δ>1; the factor allocation parameter of the industry s is α; the technical efficiency of capital cost is $A_{t}$; and the unit price of labor cost is $W_{t}$. Then, the employee brand equity (BE) at time t is expressed as follows:


(3) $$\;BE=\frac{\;W_{t}L_{i,t}^{s}}{P_{i,t}^{s}Y_{i,t}^{s}}=\frac{\delta -1}{\delta \left(1-\theta_{i,t}^{s}+\lambda \right)}\left[1-\alpha {\left(\frac{A_{t}K_{i,t-1}^{s}}{Y_{i,t}^{s}}\right)}^{\frac{\delta -1}{\delta }}\right]\;$$

If equation (2) holds, when the financing constraints increase, the denominator on the right-hand side of the equation (3) increases and BE decreases. Therefore, *ceteris paribus*, the following hypothesis is proposed.

*Hypothesis 2 (H2):* Financing constraints can significantly restrain employee brand equity.

## Methodology

### Variable selection

We use the ratio of “cash paid to and for employees to total operating income” in the cash flow statement to measure the employee income and regard it as the employee brand equity (BE) from a micro perspective (Wang and Huang, 2017; Wang and Mao, 2019). Referring to Huang et al. (2020), financing constraint (SA) is a mediating variable.


(4) $$SA=-0.737\times Ln\left(Asset\right)+0.043\times Ln{\left(Asset\right)}^{2}-0.04\times Time$$

Where Asset refers to the total assets and Time refers to the age of the enterprise. Following Qiu et al., 2018, we take the digital finance index published by the Digital Finance Research Center of Peking University as our key explanatory variable. This index quantitatively depicts stage of innovation of Internet or digital finance in China, and thus, along with its different dimensions, is a good proxy for the level of FinTech development in China by industry. The control variables and descriptive statistics are shown in Table 1. Among factors not defined before, asset-liability ratio, capital intensity, return on total assets, enterprise growth (dummy variable), and nature of equity (dummy variable) are adopted as variables as in Wang and Huang (2017) and Wang and Mao (2019). Meanwhile, equity concentration is adopted from Shi et al. (2019) and measures the equity distribution of listed companies.

**Table 1:** Summary of variables.

Type of variables	Name of variables	Symbol	Descriptions
Explained	Employee brand equity	BE	Employee compensation/total operating income
Explanatory	FinTech	FIN	Digital Financial Inclusion Index of Peking University
Mediating	Financing constraints	SA	Refer to equation (4) for details
Control	Asset-liability ratio	LEV	Total liabilities/total assets
	Capital intensity	CI	Total assets/operating income
	Return on total assets	ROA	Net profit/total assets
	Enterprise growth	Growth	Equals 1 for positive total asset growth rate; otherwise, equals 0.
	Equity concentration	SC	Shareholding ratio of top ten shareholders
	Equity nature	SOE	1 for SOEs, 0 for non-SOEs

### Research model

As mentioned before, there are specific time, region, and industry effects among the mediating variables, explanatory, and explained variables. To more concretely explore how financing constraints play a specific mediating role, we refer to Xu et al.’s (2020) theoretical model and construct the following analytical model:


(5) $$\begin{array}{c} \;BE_{i,n,t}=\beta_{1}+\beta_{2}FIN_{i,n,t}+\overset{6}{\underset{k=1}{\sum }}\gamma_{k}Control_{i,n,t}\\ +\mu_{i}+v_{t}+\varphi_{n}+\varepsilon_{i,n,t} \end{array}$$

Where BE represents the employee brand equity; FIN represents FinTech; Control represents the six control variables LEV,CI,ROA,Growth,SC,andSOE; μ represents the regional fixed effect that does not change with time and industry; v represents the time fixed effect that does not change with province and industry; φ represents the industry fixed effect that does not change with time and province; and ε represents the random disturbance term. $\beta_{1}$, $\beta_{2}$, and $\gamma_{k}$ (k = 1, 2, …, 6) are all coefficients, and the subscripts i, n, and t represent province, industry, and year, respectively.

### Data source

We take balanced panel data of 31 provinces in China (including autonomous regions and municipalities directly under the Central Government, excluding Hong Kong, Macao, and Taiwan) from 2011 to 2019. This time period is chosen for two reasons. First, the only complete interval of the FinTech index we used was 2011–2019. Second, at the end of 2019, the advent of the COVID-19 pandemic may have changed some aspect or the other, which may make the previous regularity unsustainable. We hope to use this study as a pre-pandemic reference for comparison and discussion by subsequent researchers. Except for the FinTech index, all required measurement indicators for control, explanatory, and explained variables are taken from the CSMAR database. The Pearson correlation coefficient between variables is small (refer to Table 2 for details), indicating that the regression model does not suffer from collinearity. Stata 16.0 was used for data processing.

**Table 2:** Pearson correlation analysis.

Coefficient	BE	SA	FIN	LEV	CI	ROA	Growth	SC	SOE
BE	1.00								
SA	−0.11	1.00							
FIN	0.08	0.11	1.00						
LEV	−0.06	0.33	−0.02	1.00					
CI	0.07	0.02	0.01	0.01	1.00				
ROA	−0.05	−0.01	−0.04	−0.44	−0.01	1.00			
Growth	−0.05	0.07	−0.06	−0.08	0.01	0.27	1.00		
SC	−0.04	0.18	0.02	−0.09	0.01	0.17	0.16	1.00	
SOE	−0.04	0.34	−0.09	0.22	0.00	−0.05	−0.08	−0.04	1.00

BE, Employee brand equity; FIN, FinTech; SA, Financing constraints; LEV, Asset-liability ratio; CI, Capital intensity, ROA, return on total assets; Growth, enterprise growth; SC, equity concentration; and SOE, equity nature.

## Result and discussion

### Descriptive statistics for main variables

The descriptive statistics for variables are reported in Table 3. First, asset-liability ratio varies between 0.008 and 18.84, with an average value of 0.44. This indicates that the creativity of various economic units varies significantly in China. The mean values of capital intensity and return on total assets are 6.4 and 3.6, respectively, and have standard deviations of 230.3 and 10.8, respectively. This indicates that the operating efficiency and earning capacity of listed companies varies substantially, and the development of the whole market is uneven. Enterprise growth and nature of equity are dummies with mean values of 0.8 and 0.3, respectively. The former shows a significant increase in the total assets of listed companies. The latter shows that the number of non-SOEs is roughly twice that of SOEs in our sample. Finally, the mean equity concentration is 0.60, indicating that the top 10 shareholders have significant control over Chinese listed companies.

**Table 3:** Descriptive statistics for variables.

Symbol	Number of observations	Mean	Standard deviation	Minimal value	Maximal value
BE	18,126	13.9	17.3	0.1	1605.1
FIN	18,126	239.2	94.6	16.2	410.3
SA	18,126	4.4	1.9	−1.7	17.7
LEV	18,126	44.4	31.1	0.8	1883.8
CI	18,126	6.4	230.3	0.1	25084.4
ROA	18,126	3.6	10.8	−677.6	86.4
Growth	18,126	0.8	0.4	0.0	1.0
SC	18,126	59.8	16.0	1.3	101.2
SOE	18,126	0.3	0.5	0.0	1.0

BE, employee brand equity; FIN, FinTech; SA, financing constraints; LEV, asset-liability ratio; CI, capital intensity; ROA, return on total assets; Growth, enterprise growth; SC, equity concentration; and SOE, equity nature.

### Benchmark regression

We complete the estimation of the benchmark regression by following several steps. First, we test the relationship between FinTech, and employee brand equity and financing constraints separately. The results without controls in Columns (1) and (2) of Table 4, respectively show that FinTech positively and negatively affects employee brand equity and financing constraints, respectively. The result of FinTech’s statistically significant positive effect on employee brand equity at the statistical level is in line with our expectations. In comparison, the results of the negative effect of FinTech on financing constraints in a statistically significant way are in line with the findings of Huang et al. (2020) and Deng and Zhao (2022). Second, when we consider FinTech and financing constraints as explanatory variables of employee brand equity [Column (3)], financing constraints negatively affect employee brand equity. That is, the relationship between employee brand equity and corporate financing constraints is an inverse variation. Employee brand equity decreases when a firm’s financing constraint increases, and employee brand equity decreases; conversely, employee brand equity increases when a firm’s financing constraint decreases. It could be exciting news, which shows a new factor in increasing the company’s employee brand equity. Third, the coefficient of FinTech in Column (3) is slightly smaller than that in Column (1), indicating that financing constraints are indeed the mediating variable between FinTech and employee brand equity. To ensure the validity of the results, we add six control variables: LEV, CI, ROA, Growth, SC, and SOE. Columns (4–6) of Table 4 list the result. We do not report test results for individual control variables as they are not the focus of this paper. Again, the results after adding controls are qualitatively similar to our prior results, indicating their reliability.

**Table 4:** Regression results of benchmark model.

Serial number	(1)	(2)	(3)	(4)	(5)	(6)
Variable name	BE	SA	BE	BE	SA	BE
FIN	0.052^**^ (2.69)	−0.003^**^ (−3.08)	0.048^**^ (2.61)	0.0529^**^ (2.87)	−0.028^**^ (−3.13)	0.049^***^ (2.75)
SA			−1.639^***^ (−12.33)			−1.469^***^ (−8.29)
Constant	1.414 (0.30)	5.079^***^ (23.39)	9.736^**^ (2.44)	6.920 (1.47)	3.145^***^ (9.65)	11.539^**^ (2.90)
Controls	No	No	No	Yes	Yes	Yes
Regional effect	Yes	Yes	Yes	Yes	Yes	Yes
Year effect	Yes	Yes	Yes	Yes	Yes	Yes
Industry effect	Yes	Yes	Yes	Yes	Yes	Yes
Adj.*R*^2^	0.1232	0.4505	0.1416	0.1346	0.5312	0.1472
*F* Value	7.21	9.46	102.29	288.12	3285.86	1403.02
N	18,126	18,126	18,126	18,126	18,126	18,126

*t*-values are in parentheses. ^***^p < 0.01, ^**^p < 0.05, ^*^p < 0.1. Controls refer to six control variables: LEV, CI, ROA, Growth, SC, and SOE. BE, employee brand equity; FIN, FinTech; SA, financing constraints; LEV, asset-liability ratio; CI, capital intensity; ROA, return on total assets; Growth, enterprise growth; SC, equity concentration; and SOE, equity nature.

### Robustness test

To ensure the robustness of our results, we test the possible adverse effects of endogeneity and outlier problems on the primary estimation results.

#### Endogeneity test

We use the one-period lag term of the independent variable as another independent variable in our estimation to test any contemporaneous correlation of the residual terms. The estimation equation is as follows:


(6) $$\begin{array}{c} \;BE_{i,n,t}=\beta_{1}+\beta_{2}FIN_{i,n,t}+\beta_{3}BE_{i,n,t-1}\\ +\overset{6}{\underset{k=1}{\sum }}\gamma_{k}Control_{i,n,t}+\mu_{i}+v_{t}+\varphi_{n}+\varepsilon_{i,n,t} \end{array}$$

Difference (DIF) Generalized Method of Moments (GMM) and System-GMM (SYS-GMM) are two conventional approaches to dealing with dynamic panel models such as ours. However, the former is susceptible to weak instrumental variables, producing slight sample bias (Blundell and Bond, 1998; Bond et al., 2001). Researchers usually take the two-step SYS-GMM. Column (5) of Table 5 reports the results for Model (9) with SYS-GMM. The significance of Arellano-Bond AR (1) and AR (2) tests are 0.025 and 0.828, respectively, indicating that the difference of error term only has no autocorrelation, indicating that SYS-GMM is feasible. In addition, the estimated value of the SYS GMM should be between the ordinary least squares (OLS) and fixed effect estimates (Bond et al., 2001). The regression coefficient of lagged term is 0.145, which is just between the upper and lower limits of the regression coefficient (0.350, *t*-value of 75.62; 0.093, *t*-value of 22.05, respectively), indicating that the estimates are valid.

**Table 5:** Robustness estimation.

Explained variable: BE	(4)	(7)	(8)	(9)	(10)
Variable name	FE	IV-Lag	IV-Lag-R	SYS-GMM	Outliers
FIN	0.053^**^ (2.87)	0.008^***^ (10.06)	0.019^**^ (2.76)	0.007^***^ (4.94)	0.024^***^ (4.25)
L.BE				0.145 (1.29)	
Constant	6.920 (1.47)	14.454^***^ (20.40)	16.706^***^ (10.39)	10.761^***^ (6.48)	11.518^***^ (8.34)
Controls	Yes	Yes	Yes	Yes	Yes
*p* value of Hansen Test				0.000	
*p* value of Difference—in—Hansen Test				0.000^a^	
				0.000^b^	
*p* value of AR (1) test				0.025	
-*p* value of AR (2) test				0.828	
N	18,126	15,477	15,477	15,477	17,764

Control group: Models (7)–(9) take the one-period lag values of the explanatory variables, and use all control variables; the SYS-GMM estimation adopts heteroskedasticity robust standard error to correct the *t*-values. The difference and level equation instrumental variable is FIN. The lag period of other variables is only applied to the instrumental variables of the difference equation and the lag orders take (1, 3). *a* and *b* are the probabilities for testing the validity of additional instrumental variables of the level and difference equations, respectively; outliers take the truncation regression of BE at 1 and 99% quantiles. *t* values are in parentheses. ^***^p < 0.01, ^**^p < 0.05, and ^*^p < 0.1. Controls refer to control variables: LEV, CI, ROA, Growth, SC, and SOE. BE, employee brand equity; FIN, FinTech; SA, financing constraints; LEV, asset-liability ratio; CI, capital intensity; ROA, return on total assets; Growth, enterprise growth; SC, equity concentration; and SOE, equity nature.

#### Outlier test

With the large sample size of China’s stock market and the diverse characteristics of enterprise development, greater heterogeneity in the employee brand equity may result in greater effect of outliers on the results. Therefore, we winsorize BE data at the 1 and 99% quantiles and conduct the fixed effect test again. Table 5 shows the result of the model (10). The coefficient of FinTech remains predominant, showing the reliability of previous estimates.

### Further study

Since the digital inclusive finance index has strong spatial agglomeration and heterogeneity (Guo et al., 2020), we classify it based on two dimensions: the nature of property right and regional economic heterogeneity. We obtained some interesting insights (Table 6). First, SOEs’ financing constraints have significantly weaker inhibitory effect on the labor income share than that for non-SOEs. This may be due to differences in the industry environment for these two types of enterprises. Specifically, due to the further implementation of China’s “deleveraging” reforms, the scale of lending of state-owned commercial banks has gradually tightened since 2018. Simultaneously, China’s economic situation improved, and the size and number of private enterprises grew markedly. Consequently, there was a massive gap in market financing. In addition, the financing environment also differs for SOEs and non-SOEs. Non-SOEs are subject to significant credit and budget constraints and find it difficult to raise sufficient funds from the financial sector to deal with short-term debt challenges. Then, they alleviate current liquidity problems by reducing labor costs. By contrast, state-owned enterprises may have few such concerns.

**Table 6:** Further research.

BE	Northeast China		Western Region		Eastern China		Central China	
	SE	PE	SE	PE	SE	PE	SE	PE
FIN	0.077	−0.223	0.008	0.080^**^	0.066^***^	0.033^**^	−0.052^**^	0.000
	(0.38)	(−1.65)	(0.13)	(2.65)	(5.28)	(2.72)	(−2.44)	(−0.01)
SA	−0.981	−2.131^***^	−0.923	−1.876^***^	−1.056^***^	−1.297^***^	−0.748^***^	−1.863^***^
	(−1.17)	(−4.82)	(−1.16)	(−5.07)	(−11.84)	(−13.62)	(−4.00)	(−6.77)
Controls	Yes	Yes	Yes	Yes	Yes	Yes	Yes	Yes
*R* ^2^	0.558	0.566	0.547	0.970	0.424	0.244	0.772	0.546
*N*	364	392	740	1,176	3,981	9,242	906	1,319

*t* value in parentheses. ^***^p < 0.01, ^**^p < 0.05, and ^*^p < 0.1. Controls refer to control variables: LEV, CI, ROA, Growth, SC, and SOE. SE, state-owned enterprise; PE, private enterprise; BE, employee brand equity; FIN, FinTech; SA, financing constraints; LEV, asset-liability ratio; CI, capital intensity; ROA, Return on total assets; Growth, enterprise growth; SC, equity concentration; and SOE, equity nature.

Second, the financing constraints have the most significant restraining effect on employee brand equity for private enterprises in the eastern region, followed by the central, western, and northeast region. This may because the eastern region is highly market-oriented, with less macro intervention and weaker financial repression for listed companies. Under intensifying financing constraints, shareholders from other regions may take more market risks when compared to the eastern region. Shareholders may be the biggest beneficiaries if the factors shift. For example, FinTech development has eased the financing constraints of the enterprise. In addition, the number of listed companies in the eastern region far exceeds that in other regions, indicating that the eastern coastal region has more choices than the latter in consumption and allocation of social resources.

Third, the influence behavior of FinTech on employee brand equity shows regional heterogeneity. While FinTech development significantly promotes employee brand equity for all enterprises in the eastern region, this phenomenon is only observed for private enterprises in the western region. Rather, for SOEs in the central region, it even has a negative effect. This may be because SOEs have many financing channels. For example, SOEs may enjoy preferential policies in terms of finance and taxation. Therefore, FinTech’s on SOEs’ economic activities may be limited. Besides, FinTech can break the information barrier between enterprises and financial institutions. This may greatly alleviate financing constraints for private enterprises, who generally suffer from a lack of credit as lenders find it difficult to effectively identify potential external default risks associated with private enterprises (Liu et al., 2019). Moreover, due to their political goals and economic interests, SOEs may prefer productive activities with short cycles and high performance. Then, FinTech development may be especially helpful for private enterprises who have long suffered from institutional discrimination. Thus, the external driving force of FinTech in technological innovation should be slightly more vital in private enterprises than in SOEs.

### Study limitations

The employee compensation incentive could reflect the identity of employees with brand equity. However, it is not a comprehensive index because it neglects other dimensions that affect brand equity, such as working environment, corporate culture, work intensity, and especially consumer identity, which is a hot topic and factor. Researchers downplay the role of these factors to highlight the unique value of compensation incentives in brand equity management within firms. In addition, the application value of FinTech in employee brand equity management is the most concerned topic in this paper. The reasonable estimation of the FinTech index is still a controversial and continuous issue. Since the index researchers apply is only a part of FinTech development in China, some ignored application limitations will reduce the suitable range and application value. Meanwhile, it is also a future research orientation.

## Conclusion

Using panel data on listed companies from 31 provinces, municipalities, and autonomous regions in China from 2011 to 2019, we find that FinTech’s development helps improve employee brand equity. This relationship is mediated by financing constraints. Specifically, FinTech’s development promotes social employee brand equity by easing financing constraints for enterprises. Furthermore, the inhibiting effect of financing constraints on employee brand equity has apparent cross-enterprise heterogeneity. Specifically, this inhibiting effect is stronger on the employee brand equity of non-SOEs than that of SOEs, while there is no significant effect on enterprises in the western and northeastern regions. The transmission effect of FinTech on employee brand equity also shows cross-regional heterogeneity. While FinTech development benefits employee brand equity for all enterprises in the eastern region, it only benefits private enterprises in the western region. Moreover, FinTech development even has a significant inhibiting effect on SOEs in the central region.

## Data availability statement

The original contributions presented in the study are included in the article/supplementary material, further inquiries can be directed to the corresponding author.

## Author contributions

LL contributed to the conception, methodology, and manuscript. ZH contributed to data curation, software, and review. XJ verified the data and methodology. All authors contributed to the article and approved the submitted version.

## Conflict of interest

The authors declare that the research was conducted in the absence of any commercial or financial relationships that could be construed as a potential conflict of interest.

## Publisher’s note

All claims expressed in this article are solely those of the authors and do not necessarily represent those of their affiliated organizations, or those of the publisher, the editors and the reviewers. Any product that may be evaluated in this article, or claim that may be made by its manufacturer, is not guaranteed or endorsed by the publisher.

## Footnotes

^1^The Financial Stability Board (FSB) proposed that financial technology is based on a series of technological innovations, such as big data, cloud computing, artificial intelligence, and blockchain, and fully applicable to six major financial fields, including payment clearing, loan financing, wealth management, retail banking, insurance, and transaction settlement. Indeed, it will be the mainstream in the financial industry in the future (Du et al., 2021).

^2^We assumed that most employees participate in the workforce to receive a paycheck. Thus, employees’ recognition of the enterprise’s brand value will be improved when they get more pay; On the contrary, the employees’ recognition will decrease when the remuneration decreases.
